# Susceptibility of Tsetse Species to *Glossina pallidipes* Salivary Gland Hypertrophy Virus (GpSGHV)

**DOI:** 10.3389/fmicb.2018.00701

**Published:** 2018-04-09

**Authors:** Güler Demirbas-Uzel, Henry M. Kariithi, Andrew G. Parker, Marc J. B. Vreysen, Robert L. Mach, Adly M. M. Abd-Alla

**Affiliations:** ^1^Insect Pest Control Laboratory, Joint FAO/IAEA Division of Nuclear Techniques in Food and Agriculture, International Atomic Energy Agency, Vienna, Austria; ^2^Institute of Chemical, Environmental and Biological Engineering, Research Area Biochemical Technology, Vienna University of Technology, Vienna, Austria; ^3^Biotechnology Research Institute, Kenya Agricultural & Livestock Research Organization, Nairobi, Kenya

**Keywords:** Hytrosaviridae, hypertrophy, host range, virus transmission, Glossinidae

## Abstract

Salivary gland hytrosaviruses (SGHVs, family *Hytrosaviridae*) are non-occluded dsDNA viruses that are pathogenic to some dipterans. SGHVs primarily replicate in salivary glands (SG), thereby inducing overt salivary gland hypertrophy (SGH) symptoms in their adult hosts. SGHV infection of non-SG tissues results in distinct pathobiologies, including reproductive dysfunctions in tsetse fly, *Glossina pallidipes* (Diptera: Glossinidae) and house fly. Infection with the *G. pallidipes* virus (GpSGHV) resulted in the collapse of several laboratory colonies, which hindered the implementation of area wide integrated pest management (AW-IPM) programs that had a sterile insect technique (SIT) component. Although the impact of GpSGHV infection has been studied in some detail in *G. pallidipes*, the impact of the virus infection on other tsetse species remains largely unknown. In the current study, we assessed the susceptibility of six *Glossina* species (*G. pallidipes*, *G. brevipalpis, G. m. morsitans, G. m. centralis*, *G. f. fuscipes*, and *G. p. gambiensis*) to GpSGHV infections, and the impact of the viral infection on the fly pupation rate, adult emergence, and virus replication and transmission from the larval to adult stages. We also evaluated the ability of the virus to infect conspecific *Glossina* species through serial passages. The results indicate that the susceptibility of *Glossina* to GpSGHV varied widely amongst the tested species, with *G. pallidipes* and *G. brevipalpis* being the most susceptible and most refractory to the virus, respectively. Further, virus injection into the hemocoel of teneral flies led to increased viral copy number over time, while virus injection into the third instar larvae delayed adult eclosion. Except in *G. pallidipes*, virus injection either into the larvae or teneral adults did not induce any detectable SGH symptoms, although virus infections were PCR-detectable in the fly carcasses. Taken together, our results indicate that although GpSGHV may only cause minor damage in the mass-rearing of tsetse species other than *G. pallidipes*, preventive control measures are required to avoid viral contamination and transmission in the fly colonies, particularly in the facilities where multiple tsetse species are reared.

## Introduction

The hematophagous tsetse flies (*Glossina* spp.) are responsible for transmission of African trypanosomoses, a group of anthropozoonotic neglected tropical diseases affecting humans and their livestock in most of sub-Saharan Africa (Steelman, 1976). The tsetse-infested countries are amongst the world’s least developed where hunger and poverty have been partially attributed to the presence of tsetse and trypanosomosis (Vreysen, 2006). The lack of effective vaccines and drugs against trypanosomoses makes tsetse vector control an attractive and sustainable disease management option (Leak, 1998). A promising vector control approach is the sterile insect technique (SIT), particularly when it is applied within the frame of an AW-IPM approach (Klassen and Curtis, 2005; Vreysen et al., 2013). This control tactic depends heavily on large-scale production of sterile males, which upon release into the field, out-compete the wild males in mating wild virgin females; these matings result in no offspring, which will eventually lead to a decline and eventual elimination of the target insect populations (Vreysen et al., 2000). However, the *Glossina pallidipes* salivary gland hypertrophy virus (GpSGHV; *Hytrosaviridae* family) seriously hampers mass-production of *G. pallidipes*, a competent vector of several trypanosomes (Moloo et al., 1992; Abd-Alla et al., 2010). Although the GpSGHV has not been reported to cause any significant problems in the rearing of other tsetse species, earlier studies reported SGH symptoms in natural tsetse populations of *Glossina austeni*, *G. m. morsitans*, *G. nigrofusca nigrofusca*, and *G. pallicera pallicera* (Burtt, 1945; Ellis and Maudlin, 1987; Gouteux, 1987). It is unclear whether the viral strain or isolate found in *G. pallidipes* is the same strain reported in other tsetse species. Consequently, if adequate virus management strategies are not put in place, there is a risk of the spread of GpSGHV to tsetse species other than *G. pallidipes* that are mass-produced for vector control programs that have an SIT component. As an example, some production problems were recently reported with the maintenance of *G. f. fuscipes* colonies in Bratislava, Slovakia and Addis Ababa, Ethiopia and in both facilities colonies of different tsetse species are maintained together with *G. pallidipes*. In Bratislava, the problem was very severe and resulted in the complete loss of the *G. f. fuscipes* colony, whereas in Addis Ababa, the colony size was drastically reduced from 1.3 million females to less than 100,000 flies over a period of 52 weeks. Although the reasons of the production problems with the *G. f. fuscipes* colonies are yet to be elucidated, it was deemed important to evaluate the risk of colonies of species other than *G. pallidipes* becoming infected with GpSGHV, and to clarify whether appropriate control measures will be needed to control the virus in facilities rearing multiple tsetse species. that are of specific interest for SIT/AW-IPM campaigns against tsetse and trypanosomosis.

Members of the *Hytrosaviridae* family consist of a small group of enveloped, rod-shaped dsDNA viruses that infect some dipteran insects, in which they replicate in the salivary glands (SGs) that as a result become enlarged (SGH) (Abd-Alla et al., 2009b). So far, hosts for hytrosaviruses (SGHVs) include the hematophagous tsetse fly (infected with GpSGHV), the filth-feeder housefly *Musca domestica* (infected with MdSGHV), and the phytophagous syrphid fly *Merodon equestris* (Amargier et al., 1979). Unlike in the housefly, where only one MdSGHV strain has been detected and sequenced, genomes of two GpSGHV strains/isolates have been fully sequenced (Abd-Alla et al., 2016). The two strains induce different pathobiologies in different tsetse rearing facilities based on the tsetse species, origin and domestication period (Abd-Alla et al., 2016). The observed differential GpSGHV pathologies might be attributed to genetic differences between the virus strains. Further, the pathogenesis and the transmission mechanisms of MdSGHV in the house fly differ markedly from those of GpSGHV in the tsetse fly. While horizontal transmission through *per os* infection (i.e., challenged by the development of the peritrophic membrane) is the main route for MdSGHV infection, vertical transmission from mother to offspring seems to be the main route for GpSGHV infection in natural population (Kariithi et al., 2017).

Due to the tsetse fly’s adenotrophic viviparity, GpSGHV is readily transmitted via the milk gland secretions from the mother to the developing larva (Boucias et al., 2013) and, in most cases, the virus persists in an asymptomatic infection state. GpSGHV can induce cellular hypertrophy of the SG cells (i.e., enlarged SG cells capable of replication) (Kariithi et al., 2013), which is associated with sterility in males and a reduction in the fecundity of females. Infection with GpSGHV in mass-rearing facilities occurs through feeding (*per os*) and via vertical transmission from mother to offspring (Abd-Alla et al., 2010). However, *G. pallidipes* is highly susceptible to intra-hemocoelic GpSGHV injection, which results in high viral copy numbers (≥10^9^ viral genome copies) but without either the onset of overt SGH symptoms or the release of detectable viral particles via fly saliva during *in vitro* membrane feeding (Boucias et al., 2013).

Due to the low number of SGHV strains and the limited studies conducted, little is known about the host range of these viruses. The viruses seem not to be restricted to *G. pallidipes* because earlier studies reported the occurrence of SGH symptoms in several tsetse species (Burtt, 1945; Ellis and Maudlin, 1987; Gouteux, 1987). Additionally, injection of GpSGHV into third instar larvae of *G. m. morsitans* and *G. m. centralis* induced overt infection in both the male and female adults that emerge from the virus-injected larvae (Jura and Davies-Cole, 1992; Jura et al., 1993; Sang et al., 1996, 1997; Kariithi et al., 2013). However, these previous studies did not confirm any similarity between the virus genome injected into the larvae and the virus in the adults. The occurrence of latent GpSGHV infections (Kariithi et al., 2017) makes it possible that the virus in the adults of *G. m. morsitans* and *G. m. centralis* are different latent viruses induced by the artificially injected virus. This phenomenon was previously demonstrated by the reactivation of latent *Mamestra brassicae* nuclear polyhedrosis virus (MabrNPV) infection by feeding larvae of *M. brassicae* with *Panolis flammea* NPV, which is related to MabrNPV (Hughes et al., 1993). Another study confirmed that serial passage of the virus in the insect host increases pathogenicity of the virus (Gani et al., 2014).

In the current study, to investigate the host range of the GpSGHV and its impact on other tsetse species the susceptibility of six *Glossina* species to GpSGHV infection was assessed. We also evaluated replication of the GpSGHV following intra-hemocoelic virus injection into larval and adult stages of the tested species. Dissections and quantitative polymerase chain reaction (qPCR) were used to assess the induction of latent infection and the development of overt SGH symptoms in adults emerging from virus-injected third-instar larvae and in the F_1_ progeny produced by virus-injected mothers, and to assess the potential enhancement of latent SGHV infection in conspecific larvae of each of the *Glossina* species.

## Materials and Methods

### Tsetse Species and Experimental Set-Up

The six *Glossina* species [*G. pallidipes* (Uganda), *G. brevipalpis* (Kenya), *G. morsitans morsitans* (Zimbabwe), *G. morsitans centralis* (Tanzania), *G. fuscipes fuscipes* (Central African Republic) and *G. palpalis gambiensis* (Burkina Faso)] used in this study were obtained from colonies maintained at the Insect Pest Control Laboratory (IPCL) of the Joint FAO/IAEA Division of Nuclear Techniques in Food and Agriculture, Seibersdorf, Austria. Unless otherwise stated, all experimental flies were fed on warm, defibrinated bovine blood for 10–15 min three times weekly, using an *in vitro* membrane feeding technique (Feldmann, 1994). Tsetse adults, all deposited third instar larvae and pupae were incubated at 24 ± 0.5°C until adult eclosion. Fly productivity and adult emergence were assessed using standard procedures (Feldmann, 1994).

### Virus Source and Inoculations

**FIGURE 1:**
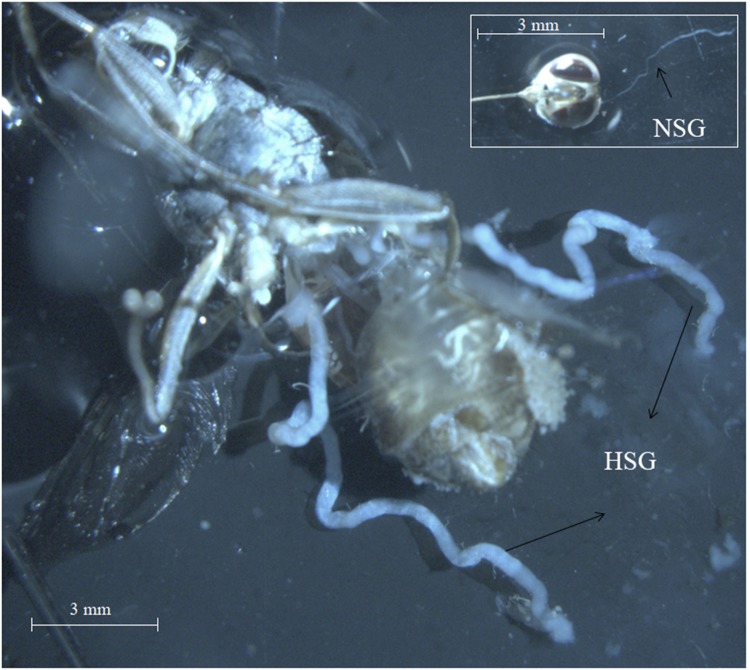
Symptoms of *Glossina pallidipes* salivary gland hypertrophy virus (GpSGHV) in tsetse fly *G. pallidipes* males with normal (NSG) and hypertrophied (HSG) salivary gland.

Salivary glands (SGs) with overt SGH symptoms were dissected from *G. pallidipes* males (**Figure 1**) and used to prepare the virus inoculum as described by Boucias et al. (2013) with slight modifications that included aseptic SG dissection and use of non-filtered virus inoculum. For conspecific virus injections, other than in *G. pallidipes*, SGs were dissected from 10-day old flies (males and females) that emerged from the larvae produced by mothers initially injected with the virus suspension derived from virus-infected *G. pallidipes*. Infection in the SGs was verified by the PCR protocol described by Abd-Alla et al. (2007). After the PCR diagnostics, the PCR-positive SG homogenates were used to prepare the virus inoculum as described above. The viral copy number was estimated with qPCR as previously described (Abd-Alla et al., 2009a).

### Virus Replication in Adults and Transmission to F_1_ Progeny

To monitor GpSGHV replication in adults, and the transmission of the virus from infected parents to their F_1_ progeny, teneral (non-fed) flies were immobilized (2–6°C for 5 min) 24 h post emergence, then maintained in a plastic Petri-dish on ice and inoculated in the thoracic cavity with either 2 μl of filter-sterilized phosphate buffered saline (PBS, control) or 2 μl of the virus suspension using a 1 ml Myjector U-40 Insulin type syringe (Teruma, Leuven, Belgium). For each tsetse species, 40 male and 120 female flies were injected and placed in standard tsetse holding cages (20 cm diameter × 5 cm height) with a density of 80 flies per cage at a 1:3 mating ratio; 2–3 replications were carried out for each species. After the injections, 8 flies (6 females and 2 males) were randomly sampled from each treatment at 0-, 1-, 3-, 5-, 7-, and 9-days post injection (dpi), and subsequently frozen at -20°C until further analyses.

### Extraction of Total DNA and PCR Amplifications

After each sampling time-point, the remaining flies (*n* = 112) were maintained under standard rearing conditions for 120 days and collected pupae were incubated until emergence of the F_1_ adults. All flies that remained alive at the end of the 120-day experimental period were dissected to assess for SGH symptoms. The F_1_ flies that emerged from the collected pupae were reared until they were 10 days old, after which 8 flies from each treatment were randomly selected and frozen at -20°C until further analyses. Total DNA of individual flies was extracted from the samples collected in the parental and F_1_ generations using the DNeasy tissue kit (QIAGEN Inc., Valencia, CA, United States) following the manufacturer’s instructions. Viral copy numbers were estimated using pooled genomic DNA (6 females and 2 males). For each DNA pool, the DNA concentration in each individual fly was determined using a spectrophotometer (Nanodrop-Synergy H1 Multi-Mode Reader, BioTek Instruments, Inc., Winooski, VT, United States), followed by dilution to ensure that all individual samples contained equal final DNA concentrations. Then, 30 μl of each diluted DNA sample was pooled and used to quantify viral copy numbers by qPCR (Abd-Alla et al., 2009a) at 0-, 1-, 5, and 9-dpi; the tsetse *β-tubulin* gene was used as a housekeeping gene to normalize the qPCR reactions. The primers and the PCR condition are given in Supplementary Table 1.

### Impact of GpSGHV on Survival and Productivity of Injected Adults

To assess the impact of virus infection on fly survival and productivity, PBS- and virus-injected teneral females and males were kept together for mating (Gooding et al., 1997). Pupal production by injected females was monitored weekly for 12 weeks, and fly productivity (number of pupae per female per 10 days, p/f/10d) calculated. The total percentage weekly mortality of the adult flies was recorded and pupae that did not emerge by day 35 of incubation were considered dead, and were therefore discarded. The emerged F_1_ flies were sexed and reared for 10 days post emergence, after which the prevalence of SGH symptoms and viral copy numbers were assessed as described above.

### Virus Replication in the Pupal Stage and Transmission to the Adult Stage

To monitor virus replication during the transition of larvae into adults, freshly deposited third instar larvae were injected with either PBS or virus suspension using a modified protocol previously described by Jura et al. (1993). Briefly, larvae were immobilized at 4°C for 1 min and then injected with 1 μl of PBS or virus suspension using a 100-μl NanoFil syringe (World Precision Instruments, Inc., Sarasota, FL, United States) equipped with a 35-gauge beveled needle. The needle was accurately placed 1 mm away from the two larval polypneustic lobes. Correct injection was verified by observing blanching of the larva. Larvae were then placed in plastic dishes over ice for 1 min to allow wound-healing and subsequently allowed to pupate (in this manuscript pupate and pupation refer to pupariate and pupariation) for 2 h at room temperature. Successful pupation rates were assessed 24 h post larval-injection; pupae were incubated at 25 ± 0.5°C until adult emergence. Ten days post adult eclosion, all flies were assessed for the occurrence of SGH symptoms by microscopy during SG dissections. The total DNA was extracted from the fly carcasses, and the SGs were homogenized in PBS (1 pair of SG/100 μl PBS) and assayed for virus presence by PCR (Abd-Alla et al., 2007).

### Impact of GpSGHV Infection on the Induction of SGHV Latent Virus in Conspecific Tsetse

To assess the impact of GpSGHV infection on the possible induction of latent SGHV infections in other tsetse species, PCR-positive SG were collected from 10-day old adults (other than *G. pallidipes*) that had emerged from larvae that were injected with GpSGHV and a virus suspension was prepared as described above. The virus suspension collected from each tsetse species, positive control (SGHV collected from *G. pallidipes*), and negative control (PBS) were injected into third instar larvae of the same species. Larvae that pupated were maintained until adult emergence and adults were maintained for 10 days and then dissected to assess the SGH status and the SGs were tested by PCR as described above. The observed emergence rates in the GpSGHV or viremic SG homogenate injected larvae were corrected with Abbott’s formula (Abbott, 1925).

### Statistical Analysis

The significance of the overall differences of the virus copy numbers obtained from the various treatments were assessed by ANOVA (Sokal and Rohlf, 2012), and the significance of differences between the group’s means (PBS vs. virus injections, and the six *Glossina* species) was determined by Tukey’s honestly significant difference (HSD) test. The analyses were done in R (R Development Core Team, 2017) using RStudio (RStudio, 2016).

## Results

### Susceptibility of Different Glossina Species to GpSGHV Infections

**FIGURE 2:**
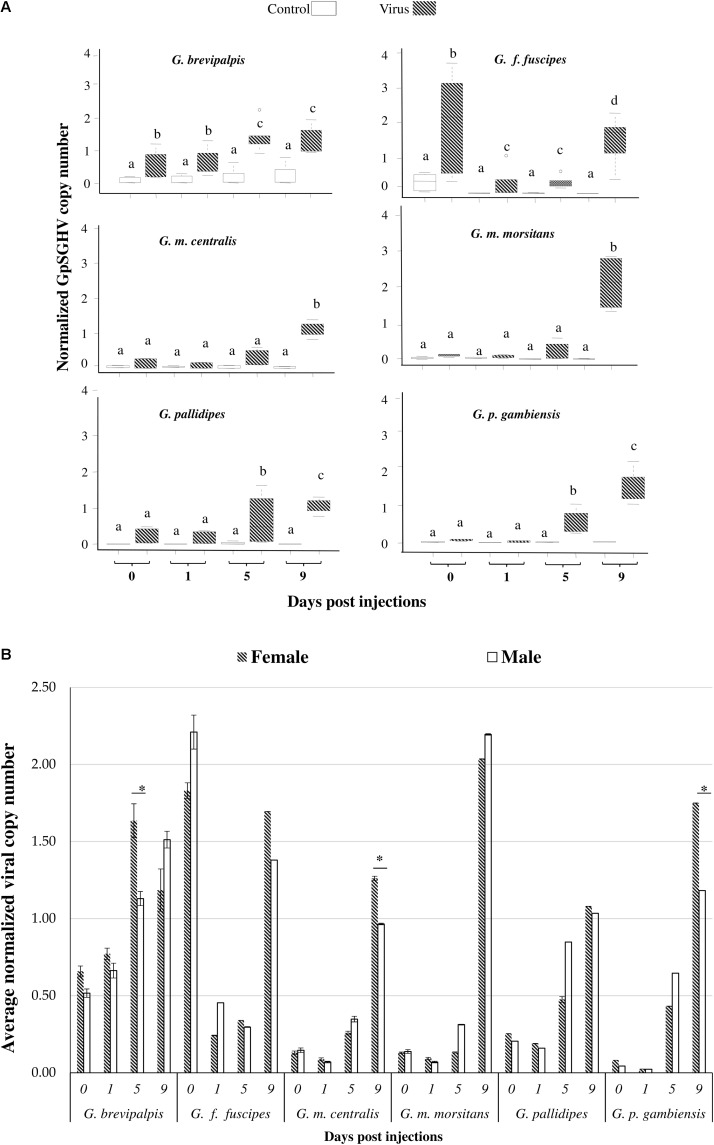
Susceptibility of *Glossina* species to GpSGHV infection. **(A)** GpSGHV copy number of six tsetse species. **(B)** Difference in GpSGHV copy number between males and females. Teneral adults from the six *Glossina* species were injected (intra-hemocoelic) with GpSGHV suspension and PBS, Viral copy numbers were quantified by qPCR from flies sampled from each species at 0, 1, 5, and 9 days post adult emergence. The qPCR data were normalized against the tsetse housekeeping gene, *β-tubulin*. Different letters in **A** and star(^∗^) in **B** indicate significant difference at the *p* = 0.05 level (Tukey HSD at the 95% family wise confidence level).

The intra-hemocoelic virus injections into teneral female and male adults showed that all six tsetse species were susceptible to GpSGHV infection (**Figure 2A**). The baseline viral copy number of the PBS-injected flies remained relatively stable over the 0–9 dpi period. Except in *G. f. fuscipes*, the viral copy number increased significantly in virus injected flies over the 0–9 dpi period for all tested *Glossina* species (df = 3, 36; *F* = 63.2; *P* < < 0.001) (**Figure 2A** and Supplementary Figure 1), but virus copy number varied significantly between the different tsetse species (df = 5, 330; *F* = 3.92; *P* < 0.05). There was no significant difference in susceptibility to virus infection between female and male flies for all tsetse species (df = 1, 35; *F* = 0.95; *P* > 0.05) (**Figure 2B**). Unlike in the other species, the viral copy number in *G. brevipalpis* increased between 0 to 5 dpi (df = 1, 6; *F* = 45.19; *P* < 0.001) (**Figure 2A**). The virus copy number in *G. pallidipes* increased as of 5 dpi, (**Figure 2A**), but in the case of *G. m. centralis* and *G. m. morsitans*, viral copy numbers only increased at 9 dpi. Comparing the overall increase in the viral copy number during the 0–9 dpi period of the six species, *G. p. gambiensis* males showed the highest increase in viral copy number, followed by *G. m. morsitans* males and *G. p. gambiensis* females. Despite this temporal increase in viral copy numbers, dissection of the SGs at the end of the experimental period (120 dpi) showed no evidence of overt SGH symptoms in any of the six *Glossina* species.

### Impact of GpSGHV on the Survival and Productivity of Female Flies and Their F_1_ Progeny

#### Survival and Productivity of Injected Female Flies

**FIGURE 3:**
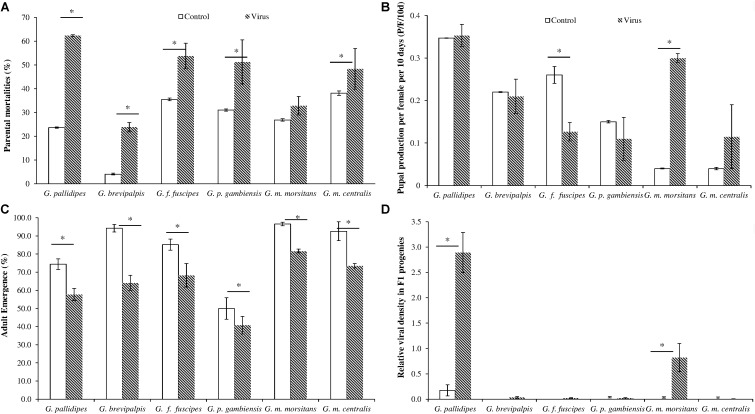
Impact of GpSGHV infection on fly survival and productivity. Teneral females were injected with GpSGHV suspension or PBS and mated with healthy males. Mortality **(A)** and pupal production per female per 10 days (p/f/10d) **(B)** were monitored weekly for 12 weeks. The rates of adult emergence and the prevalence of virus infections in the F1 progeny are shown in **(C,D)**, respectively. The qPCR quantification data on viral copy numbers were normalized against the tsetse housekeeping gene, *β-tubulin*. ^∗^Indicate significant difference at the *p* = 0.05 level (Tukey HSD at the 95% family wise confidence level).

Cumulative data over the 120-day experimental period revealed that injecting the virus in adults significantly increased mortality in both females and males compared with the untreated controls for all six *Glossina* species (df = 1, 20; *F* = 73.50; *P* < < 0.001) (**Figure 3A**). The highest and lowest virus-induced mortality was recorded in *G. pallidipes* (62% compared to 23% in the controls) (df = 1, 4; *F* = 10136; *P* < < 0.001), and in *G. brevipalpis* (23.8% compared to 4% in the controls) (df = 1, 4; *F* = 102.5; *P* < < 0.001), respectively (**Figure 3A**). The virus-induced mortality was similar in *G. f. fuscipes*, *G. p. gambiensis* and *G. m. centralis* (i.e., 53.7, 51.2, and 48.3%, respectively). Injecting the virus in adult (female and male) flies significantly reduced (df = 1, 4; *F* = 37.2; *P* < 0.05) pupal production in *G. f. fuscipes* (p/f/10d) compared to non-injected control flies (**Figure 3B**), but not in *G. pallidipes* (df = 1, 4; *F* = 0.1831; *P* > 0.05), *G. brevipalpis* (df = 1, 4; *F* = 0.0612; *P* > 0.05), *G. p. gambiensis* (df = 1, 4; *F* = 0.6316; *P* > 0.05) and *G. m. centralis* (df = 1, 2; *F* = 0.5378; *P* > 0.05). Virus injection significantly increased pupal production in *G. m. morsitans* (df = 1, 2; *F* = 676; *P* < 0.05), but this requires further investigation (**Figure 3B**).

#### Emergence and Virus Prevalence in F1 Progeny

In general, a significant difference was observed in the adult emergence rate among different virus-injected tsetse species (df = 5, 12; *F* = 3028.7; *P <* < 0.001). Emergence of F_1_ descendants from pupae produced by virus-injected female parents was reduced significantly as compared with the PBS injected control flies across all six species (df = 5, 24; *F* = 278; *P* < < 0.001) (**Figure 3C**). The control groups showed high emergence rates (>75%) for all tested species, except for *G. p. gambiensis* (50%), which had lower emergence rates than the F_1_ adult emergence from virus-injected mothers of other species. Although virus injection in adult *G. brevipalpis* induced the lowest parental mortality rates as compared with other species (**Figure 3A**), the injected virus had more impact on the emergence rate of the F_1_ progeny as compared with the untreated controls, i.e., 64.2% versus 95.2% in the control groups (**Figure 3C**). Viral copy number was high enough to be measurable by qPCR in the F_1_ adults produced by the virus-injected *G. pallidipes* and *G. m. morsitans* flies, whereas only background viral copy numbers were detected in the control flies and in the progenies produced by virus-injected mothers of the other four tsetse species (**Figure 3D**). There were no detectable SGH symptoms in any of the F_1_ progeny across all the species except *G. pallidipes* (41.9%).

### GpSGHV Injection in Larvae and Adult Emergence, Virus Transmission and Replication in Adult Flies

#### Adult Emergence of Injected Larvae

The third instar larvae that successfully pupated after injection were incubated for adult emergence. Compared to the expected normal pupal period for *Glossina* spp., adult emergence was delayed by 3–5 days in all six species irrespective of whether the larvae had been injected with PBS or virus (data not shown). The impact of virus injection on adult eclosion is presented in **Table 1**. Generally, adult emergence rates between the PBS and the virus injected larvae or between the different species injected with the virus were not significantly different (df = 1, 24; *F* = 2.72; *P* > 0.05 and df = 5, 12; *F* = 1.711; *P* > 0.05, respectively). However, *G. brevipalpis* showed the largest difference in the average adult emergence rate between the PBS and virus-injected flies (74.7 and 24.4% respectively), whereas the differences in *G. m. centralis* and *G. m. morsitans* were smaller (39.23% versus 26.54% and 60.97% versus 48.47%, respectively) (**Table 1**).

**Table 1:** Rate of adult emergence and prevalence of SGHV symptoms in adult flies that emerged from PBS- and virus injected larvae.

Species	Treatment	*N*	Adult emergence (%)	Prevalence of the SGH symptoms in the F_1_ progeny		
				By dissection	PCR analysis	
					SGs	Carcass (negative SGs)^†^
*G. p. gambiensis*	PBS	366	41.5	0/32 (0.0)	0/32 (0.0)	0/16 (0.0)
	GpSGHV	357	42.8	0/153 (0.0)	25/153 (16.3)^b,c^	11/16 (68.7)^b^
*G. m. centralis*	PBS	209	39.2	0/32 (0.0)	0/32 (0.0)	0/16 (0.0)
	GpSGHV	358	26.5	0/95 (0.0)	7/95 (7.3)^a,b^	9/45 (20.0)^a^
*G. pallidipes*	PBS	172	51.7	2/32 (6.2)	2/32 (6.25)	0/16 (0.0)
	GpSGHV	290	54.4	95/158 (60.1)	95/158 (60.12)^d^	32/32(100.0)^c^
*G. m. morsitans*	PBS	269	60.9	0/32 (0.0)	0/32 (0.0)	0/16 (0.0)
	GpSGHV	262	48.4	0/127 (0.0)	9/127(7.08)^a,b^	39/41 (95.1)^c^
*G. f. fuscipes*	PBS	294	40.8	0/120 (0.0)	0/32 (0.0)	0/24 (0.0)
	GpSGHV	207	41.0	0/85 (0.0)	21/85 (27.1)^c^	3/17 (17.6)^a^
*G. brevipalpis*	PBS	87	74.7	0/65 (0.0)	0/32 (0.0)	0/16 (0.0)
	GpSGHV	250	24.4	0/61 (0.0)	0/61 (0.0)^a^	12/12 (100.0)^c^

*^†^The numbers within one column followed by the same lower-case letters do not differ significantly at the p = 0.05 level (Tukey HSD test at the 95% family wise confidence level). N, number of successfully pupated larvae.*

#### Virus Replication and Occurrence of SGH Symptoms in Adult Flies Emerged From GpSGHV Injected Larvae

Salivary glands were dissected on day 10 post-emergence from males and females that had developed from PBS- and virus-injected larvae to assess the occurrence of the SGH symptoms (**Table 1**). SGH symptoms were observed in 6.2 and 60.1% of adult *G. pallidipes* that developed from the PBS-injected and the virus-injected larvae, respectively. Dissection results for the other five species were negative for SGH symptoms. In addition to the fly dissections, PCR analyses were carried out on all dissected SGs and their corresponding carcasses to assess GpSGHV infections (**Table 1**). The frequency of virus infections detected by PCR of the SG homogenates of all species showed large variations and was significantly different (df = 5, 12; *F* = 99.3; P < < 0.001) among different species with the highest virus infection rate in *G. f. fuscipes* and no virus detected in G. brevipalpis (**Table 1**). Virus was detectable by PCR in the fly carcasses of all the flies that emerged from the virus-injected larvae, but without overt SGH symptoms. The infections were generally higher in the carcasses than in the SG homogenates. For instance, whereas 100% of *G. pallidipes* carcasses were PCR- positive, only 60.1% of the dissected SGs homogenates were PCR positive for virus infection. Furthermore, 37.5% of the *G. f. fuscipes* carcasses of the adults that emerged from the PBS-injected larvae had detectable virus infections. Whether this result is a case of virus reactivation from a latent state requires further investigations.

### Impact of GpSGHV Induction of SGHV Latent Infection in Conspecific Glossina Species

**FIGURE 4:**
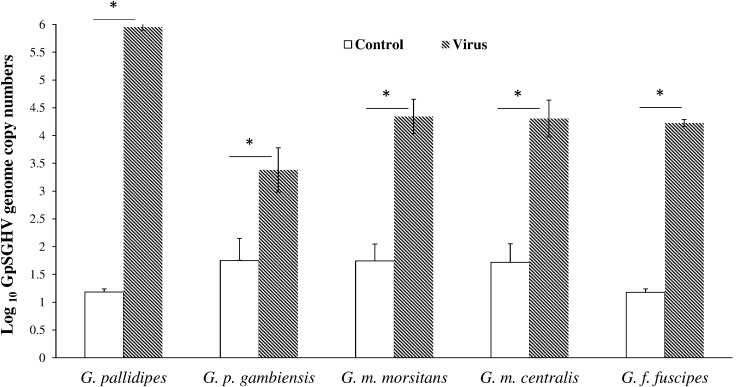
Viral copy numbers in conspecific *Glossina* species. Absolute viral copy numbers in the homogenates of the SGs dissected from 10-day old flies. These SG homogenates from the viremic flies were used to inject newly larviposited conspecific third instar larvae. ^∗^Indicate significant difference at the *p* = 0.05 level (Tukey HSD at the 95% family wise confidence level).

We investigated the impact of GpSGHV infection on the induction of SGHV latent infection and the possibility of enhancing GpSGHV infection through passaging the virus in conspecific tsetse. For this we prepared SG homogenates dissected from 10-days-old adults that emerged from virus-injected larvae that were positive for virus by PCR, and re-injected the virus suspensions into conspecific third-instar larvae. The viral copy number in 10-days old adults emerged from virus injected larvae varied significantly among species (df = 4, 10; *F* = 94,4; *P* < < 0.001), with the highest viral genome copy number recorded in *G. pallidipes* (∼10^5.9^ copies) and the lowest in *G. p. gambiensis* (∼10^3.7^ copies) (**Figure 4**). There were no detectable viral infections in the SGs dissected from 10-day-old *G. brevipalpis* that were used for the conspecific injections; *G. brevipalpis* was therefore not included in the conspecifics bioassays.

#### Adult Emergence of Flies Developed From Larvae Injected With SG Homogenates Derived From Conspecifics

When the SG homogenates were re-injected into conspecific third instar larvae, the rates of adult emergence are presented in **Table 2**. No significant differences were observed among different species (df = 4, 10; *F* = 1.031; *P* > 0.05), or between the emergence rate of the GpSGHV and conspecific injected groups (df = 5, 24; *F* = 1.68; *P* > 0.05) (**Table 2**).

**Table 2:** Rate of adult emergence and prevalence of SGHV symptoms in conspecific *Glossina* species that emerged from PBS-, GpSGHV-, and Virus-injected larvae of different tsetse species.

Species	Treatment	*N*	Adult emergence (%)	Prevalence of the SGH symptoms in the F_1_ progeny		
				By dissection	PCR analysis	
					SGs	Carcass (PCR-negative SGs)^†^
*G. pallidipes*	PBS	139; (53.6)	50.3	0/70 (0.0)	0/70 (0.0)	0/16 (0.0)
	GpSGHV	184; (63.0)	22.8	25/42 (59.5)	25/42 (59.5)^a^	15/16 (93.7)^a^
*G. p. gambiensis*	PBS	155; (49.8)	60.0	0/93 (0.0)	0/93 (0.0)	0/16 (0.0)
	GpSGHV	250; (63.9)	52.8	0/132 (0.0)	24/132 (18.8)^c^	4/8 (50.0)^b,c^
	Conspecific	313; (68.4)	66.4	0/208 (0.0)	0/208 (0.0)	1/16 (6.25)^b^
*G. m. centralis*	PBS	67; (50.0)	56.7	0/38 (0.0)	0/38 (0.0)	0/16 (0.0)
	GpSGHV	92; (52.8)	58.7	0/54 (0.0)	5/54 (9.2)^d^	7/11 (63.6)^a,c^
	Conspecific	183; (75.3)	46.4	0/85 (0.0)	2/85 (2.3)	0/14 (0.0)^b^
*G. m. morsitans*	PBS	82; (50.9)	84.1	0/69 (0.0)	0/69 (0.0)	0/16 (0.0)
	GpSGHV	131; (62.3)	54.2	0/71 (0.0)	7/71 (9.8)^d^	6/9 (66.6)^a^
	Conspecific	124; (80.0)	33.8	0/42 (0.0)	0/42 (0.0)	8/16 (50.0)^a^
*G. f. fuscipes*	PBS	130; (50.9)	46.1	0/60 (0.0)	0/60 (0.0)	0/16 (0.0)
	GpSGHV	259; (68.7)	47.4	0/123 (0.0)	30/123 (24.3)^b^	4/14 (28.5)^b^
	Conspecific	114; (62.9)	52.6	0/60 (0.0)	0/60 (0.0)	9/16 (56.2)^a^

*^†^The numbers within one column followed by the same lower-case letters do not differ significantly at the p = 0.05 level (Tukey HSD at the 95% family wise confidence level.). N, number of successfully pupated larvae.*

#### Virus Infections in Adults That Developed From Larvae Injected With SG Homogenates Derived From Conspecifics

When SGs were dissected from 10-day old flies, overt SGH symptoms were only detectable in *G. pallidipes* flies that developed from larvae injected with virus homogenates prepared from hypertrophied SGs of *G. pallidipes* (**Table 2**). When the dissected SGs were subjected to conventional PCR, virus infections were detected in the glands dissected from flies injected with virus suspensions prepared from the hypertrophied SGs of *G. pallidipes*. Viral prevalence was highest (93.7%) in *G. pallidipes* and was lowest in *G. m. centralis* (9.2%) (**Table 2**). The difference in the viral prevalence amongst the tested species was significant (df = 4, 10; *F* = 124,77; *P* < < 0.001) (**Table 2**). Following conspecific injection, virus infection was detectable only in the SGs dissected from *G. m. centralis* (2.3%). As it is possible that injected GpSGHV does not reach and replicate in fully differentiated tsetse SGs (Boucias et al., 2013), we analyzed the carcasses of the flies that did not show virus infections in the dissected SGs. Virus infections were detectable in all species in the flies that emerged from larvae injected with virus suspensions prepared from hypertrophied SGs of *G. pallidipes* (**Table 2**). The prevalence of virus infections varied significantly among the tested species (df = 4,10; *F* = 11.366; *P* < < 0.001) and was highest in *G. pallidipes* (93.7% of the analyzed individuals), but virus infections in the other species were also common; 66.6, 63.6, 50, and 28.5% in *G. m. morsitans*, *G. m. centralis*, *G. p. gambiensis*, and *G. f. fuscipes*, respectively. Following conspecific injection, virus infections were detectable in the carcasses of *G. f. fuscipes* (56.2%), *G. m. morsitans* (50%) and *G. p. gambiensis* (6.25%) (**Table 2**).

## Discussion

The results of this study demonstrate that all tested tsetse species can become infected, although at different levels, with the GpSGHV by injecting the adult flies or the third instar larvae, and the virus can replicate itself in these flies. However, SGH symptoms were only observed in virus-infected *G. pallidipes* and not in the other tested species. Similar observations were made with the *Musca domestica* MdSGHV, which can infect stable flies (*Stomoxys calcitrans*) and black dump flies (*Hydrotaea aenescens*), but does not induce SGH symptoms in the heterologous hosts (Geden et al., 2011).

Our data indicate a GpSGHV-induced increase in fly mortality and reduction in pupal productivity and emergence in all species tested, despite the absence of SGH symptoms. These negative effects of virus presence on colony performance of all tsetse species tested may affect the efficiency and cost efficacy of SIT application. Although it is unlikely that virus presence in colonies of these species jeopardizes colony stability as it does in *G. pallidipes*, it will make the mass-rearing process more tedious and expensive, i.e., a slower rate of increase in colony size and the need to maintain more females in the colony to produce the same number of sterile males.

The failure to detect overt SGH symptoms in any of the adult flies or third instar larvae (except in *G. pallidipes*) that were virus-injected agrees with previous reports (Abd-Alla et al., 2007; Boucias et al., 2013) but contradicts previous results with *G. m. centralis* and *G. m. morsitans* (Jura et al., 1993; Sang et al., 1997). The observed difference between our and previous results may be attributed to differences in the virulence of the viral strain. It is possible that the GpSGHV strain used in our study was less pathogenic than the strain(s) used in the earlier studies. Alternatively, the tsetse colonies used in our study have been cultured for more than 2 decades and it is possible that the flies derived from these colonies have become more adapted to the virus compared with the tsetse strains used in the studies in the 1990’s.

It was previously demonstrated that intra-hemocoelic injection of GpSGHV in adult flies did not cause the development of SGH symptoms but lead to SGH development in the F_1_ offspring. It was, therefore, concluded that the virus infection requires element(s) from undifferentiated tissues to induce SGH symptoms (Boucias et al., 2013). In view of this, it was anticipated that intra-hemocoelic injection of GpSGHV into the third instar larvae might lead to the development of SGH in adult flies of the tested species. SGH was, however, only observed in *G. pallidipes* adult that emerged from virus-injected larvae and the absence of SGH in other species seems to indicate the existence of additional barriers that hamper the development of SGH. Perhaps a delay in virus replication prevented the threshold of 10^6^ virus copies being reached that is required to cause SGH. Although a lower virus copy number might be a cause of the absence of SGH, in the virus-injected adults of *G. pallidipes* more than 10^6^ virus copy numbers were obtained but without occurrence of overt SGH symptoms. This might indicate that virus replication and transmission from infected pupa to adult plays a major role in the development of SGH; the detection of SGH in 1-day old *G. pallidipes* adults that emerged from pupae produced by virus injected mothers is clear evidence of virus replication in the pupal stage (Boucias et al., 2013). The absence of SGH in tsetse species other than *G. pallidipes* injected as third instar larvae might be due to unknown challenges that block virus replication in the pupae, reducing the virus copy numbers needed to induce SGH in emerged adults. Viral copy number in the surviving pupae was not assessed after injecting larvae to investigate this point and this will be analyzed in a further study. Differences in virus copy number, mortality rate and productivity in adults injected with the GpSGHV or in adult emergence rate, virus copy number or virus infection rate in both virus-injected adults and larvae of the different tsetse species may be due to the species biology and associated microbiota.

Although the GpSGHV-induced mortality and the reduced productivity observed in the injected adults of different *Glossina* species was not surprising, it is unknown how viral infection results in host mortality. However, our results agree with previous reports on the increased mortality rate of wild *G. pallidipes* infected with GpSGHV (Jaenson, 1986). In general, virus infections in insects are often associated with various biological costs, such as reduced growth/development rates and productivity (Cabodevilla et al., 2011). Host insects generally respond to pathogen infection by reduction of cellular metabolism (cessation of the synthesis and turnover of macromolecules) and cellular signaling, amongst other processes (e.g., transcription and translation) (Hand and Hardewig, 1996). If the virus pathogenesis progresses, this metabolic depression could lead to programmed cell death (apoptosis) (Sparks et al., 2008), which in turn severely affects viral gene expression, DNA replication and production of progeny virus. Consequently, it is possible that the range of hosts in which a certain virus can replicate is influenced by the ability of host insect cells to commit suicide during virus infection. Together, these facts could partially explain the differential virus-induced mortalities observed amongst the six *Glossina* species in the current study. It is difficult to explain why this high mortality was not observed in virus-injected *G. brevipalpis* but it might be related to its larger body size as the same amount of virus inoculum was injected in all flies, resulting in a relative lower virus concentration per unit weight in *G. brevipalpis* compared to other species.

The observed significant reduction in F_1_ adult emergence produced by the virus-infected mothers compared to their PBS-injected counterparts across all six *Glossina* species is most probably due to the biological cost of the virus infection. It is noteworthy that, although virus injection did not show high parental mortalities in *G. brevipalpis* (unlike in the other species) compared to the controls, the virus caused the greatest reduction in F_1_ adult emergence in this species. Virus infection could interfere with larval to pupal metamorphosis in several ways, including neuroendocrine regulation of hormonal synthesis, or transcriptional disruption of the expression of enzymes that are critical for metamorphosis. This has been demonstrated during virus infection in various Diptera such as the fruit fly, *Drosophila melanogaster* and the tobacco hornworm, *Manduca sexta* (Uhlirova et al., 2003).

The effects of GpSGHV injection into third instar larvae on pupation rate, pupal period, and adult emergence varied widely amongst the *Glossina* species. GpSGHV caused the lowest pupation rate in *G. pallidipes*, which is not surprising in view that this species seems to be the most susceptible to the virus. As the injection process was conducted in the late third instar larvae a few minutes before pupation, it is possible that the failure of the larvae to pupate was caused by the mechanical damage during injection and handling and not by the presence of the virus. The results indicate slight delay and reduction in adult emergence of *G. brevipalpis*, *G. m. morsitans*, and *G. m. centralis* from pupae that developed from GpSGHV-injected larvae. Given that there were no variations in the pupal incubation temperature and humidity, these results imply that the delayed adult emergence, at least in the above-mentioned *Glossina* spp., was due to the virus infection. Our results agree with the results obtained by Jura et al. (1993) who reported an adult emergence rate of *G. m. morsitans* after virus injections into larvae of76% compared to 85.8% in the controls. It is possible that the reduction in the emergence rate is hormonally mediated. Our study, however, did not include investigations into the ecdysteroid titers in the treated larvae.

In tsetse species other than *G. pallidipes*, the absence of SGH in 10-day old adults emerged from virus injected larvae together with the detection of the virus in some SGs and most of the fly carcasses leaves some room for speculation on the nature of the virus detected in these species. First, we hypothesize that infection with GpSGHV might just induce a latent virus infection; in such case the induced virus might be more pathogenic to the conspecific as has been reported in baculoviruses (Hughes et al., 1993). Secondly, we hypothesize that, although GpSGHV is the virus transmitted in our study in each tsetse species, the virus might not yet be adapted to these different tsetse species. The GpSGHV infection in these tsetse species might be improved through serial passages as was demonstrated also for baculoviruses (Gani et al., 2014). Taken together, the absence of enhanced virus infection in other species might indicate absence of species-specific latent virus. Instead, it is more likely that the injected GpSGHV strain was transmitted from pupae to adult. The reduced pathogenicity observed in the conspecific injection is most probably due to injecting a lower viral copy number in the conspecific compared to the virus collected from SGs. In addition, a single virus passage through heterogeneous host may be insufficient to improve GpSGHV pathogenesis (Gani et al., 2014).

In tsetse mass-rearing facilities where several tsetse species are maintained and fed on the *in vitro* membrane feeding system, due to economic reasons there is a tendency to use the same membrane for several successive feeding rounds of the same or even several species (Feldmann, 1994). It is important to note that rearing of *G. pallidipes* is in general more challenging compared with other tsetse species due to the GpSGHV infection; therefore in the case of feeding more than one species on the same membrane, it is common to feed the *G. pallidipes* flies first followed by flies of the other species. This may also be because, so far, no other species have been reported to be affected by the GpSGHV. However, this feeding protocol might have contributed to the loss of the *G. f. fuscipes* colony in Bratislava and the reduction in size of the colony maintained in Addis Ababa, Ethiopia. Additional research will be required to elucidate the reasons of the bad performance of these colonies.

## Conclusion

It should be noted that the data from our study was based on intra-hemocoelic virus injections, which is not the natural infection route for the virus. Apart from mother to offspring vertical transmission, oral infection is the primary GpSGHV infection route, which encounters several barriers (e.g., the peritrophic membrane). Compared to injection, oral infection may significantly reduce the chances of productive virus infection. The implications of these facts are that since the intra-hemocoelic injection did not induce development of overt SGH in most of the *Glossina* species analyzed in this study, it is much less likely that the natural route (via blood meal feeding) will induce the expression of overt disease symptoms. This notwithstanding, the finding that all the *Glossina* species are susceptible to GpSGHV infection and that it reduces colony performance points to the need for the implementation of strict protocols to protect colonies from GpSGHV infection. We have already developed and implemented GpSGHV management protocols that are effective in the control of the virus in *G. pallidipes* colonies (Abd-Alla et al., 2012, 2013, 2014).

## Author Contributions

GD-U, AP, HK, and AA-A conceived and designed the experiments and analyzed the data. GD-U performed the experiments. HK, GD-U, AA-A, AP, and MV wrote the paper. All the authors read and approved the final manuscript.

## Conflict of Interest Statement

The authors declare that the research was conducted in the absence of any commercial or financial relationships that could be construed as a potential conflict of interest.

## Supplementary Material

The data for this paper are available at the Harvard Dataverse through the following link (https://dataverse.harvard.edu/dataset.xhtml?persistentId=doi:10.7910/DVN/LZS5EB).
