# Supplementary material for: Susceptibility of Tsetse Species to Glossina pallidipes Salivary Gland Hypertrophy Virus (GpSGHV)
Source: Front Microbiol. 2018 Apr 9;9:701. doi: 10.3389/fmicb.2018.00701 (PMC5901070; doi:10.3389/fmicb.2018.00701)
Supplement: Supplementary file 1 [file Data_Sheet_1.PDF]

Supplementary Table 1. List of primers used for quantitative PCR (qPCR) analyses of GpSGHV and microbiome in *Glossina* species.

**Target Gene**	**Primer Name**	**Primer Sequence (Listed 5- to -3)**	**Annealing Temperature (˚C)**	**Amplicon Size (bp)**	**References**
odv-e66 (GpSGHV ORF5)	qPCRFwda	CAAATGATCCGTCGTGGTAGAA	60	51	(Abd-Alla etal.,2009;Abd-Alla et al., 2011)
	qPCRRev	AAGCCGATTATGTCATGGAAGG			
β-tubilin (Tsetse Fly)	Tsetse-tubulinF	GAT GGT CAA GTG CGA TCC T	55	355	(Caljon et al., 2009)
	Tsetse-tubulinR	TGA GAA CTC GCC TTC TTC C			

Reference List

Abd-Alla, A. M. M., Cousserans, F., Parker, A., Bergoin, M., Chiraz, J. & Robinson, A. (2009). Quantitative PCR analysis of the salivary gland hypertrophy virus (GpSGHV) in a laboratory colony of *Glossina pallidipes*. *Virus Res* **139**, 48-53.

Abd-Alla, A. M. M., Salem, T. Z., Parker, A. G., Wang, Y., Jehle, J. A., Vreysen, M. J. B. & Boucias, D. (2011). Universal primers for rapid detection of hytrosaviruses. *J Virol Methods* **171**, 280-283.

Caljon, G., Broos, K., Goeyse, I. D., De Ridder, K., Sternberg, J. M., Coosemans, M., De Baetselier, P., Guisez, Y. & Van Den Abbeele, J. (2009). Identification of a functional Antigen5-related allergen in the saliva of a blood feeding insect, the tsetse fly. 39 edn, pp. 332-341.

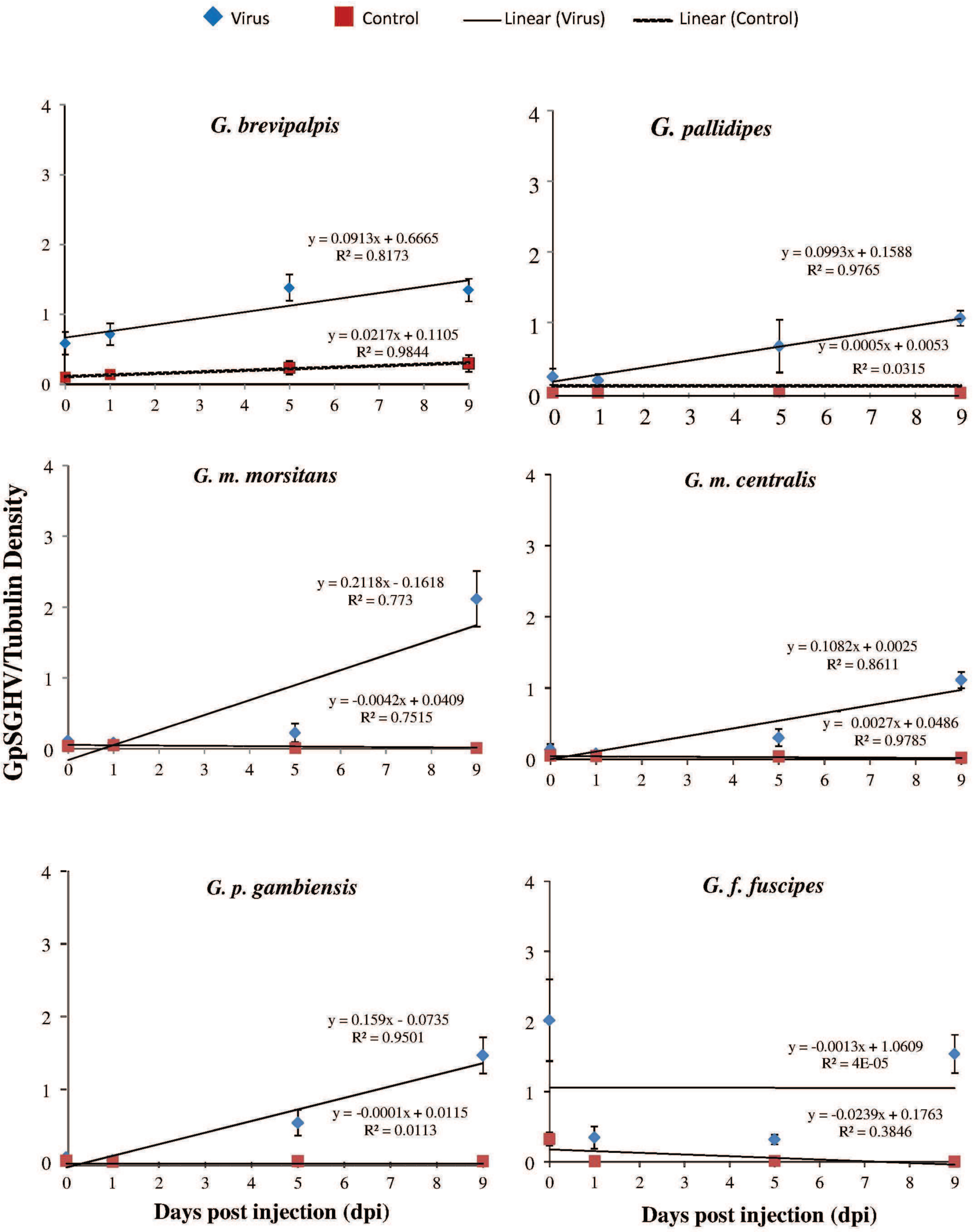

Supplementary Figure 1. Regression line of the increased virus density of injected flies
